# Identification of Critical miRNAs miR‐4652 and miR‐1304 as Novel Diagnostic Markers for Oral Squamous Cell Carcinoma

**DOI:** 10.1002/cnr2.70552

**Published:** 2026-05-01

**Authors:** Sara Bagheri Farahani, Ehsan Keramati, Marziyeh Etesami, Faranak Jamshidian

**Affiliations:** ^1^ Department of Biology ET.C., Islamic Azad University Tehran Iran; ^2^ Basic and Molecular Epidemiology of Gastrointestinal Disorders Research Center, Research Institute for Gastroenterology and Liver Diseases Shahid Beheshti University of Medical Sciences Tehran Iran; ^3^ Department of Genetics TeMS.C., Islamic Azad University Tehran Iran

**Keywords:** C‐Myc, miR‐1304, miR‐4652, oral squamous cell carcinoma

## Abstract

**Background:**

Oral squamous cell carcinoma (OSCC) is marked by frequent recurrence rates and an unclear etiology, underscoring the critical need for early detection to improve therapeutic outcomes and reduce healthcare costs. MicroRNAs (miRNAs) have emerged as key regulators of oral carcinogenesis by modulating gene expression at the posttranscriptional level and influencing various aspects of cellular physiology.

**Objective:**

This study aimed to comprehensively evaluate the prognostic significance of miR‐1304 and miR‐4652 expression levels in patients with OSCC, and to explore their potential as predictive biomarkers for disease progression and patient survival.

**Methods:**

TargetScan was used to predict potential gene interactions of the microRNAs. Subsequently, the expression levels of C‐Myc and the microRNAs miR‐1304‐3p and miR‐4652‐5p were evaluated in 30 pairs of OSCC and adjacent normal tissue samples. qRT‐PCR analyses were performed to compare the expression of these molecules between tumor and normal tissues. Additionally, receiver operating characteristic (ROC) curves were generated to assess the potential diagnostic value of these microRNAs in OSCC.

**Result:**

The expression levels of miR‐1304, miR‐4652, and C‐Myc were significantly higher in OSCC tissues compared to their matched adjacent non‐tumor tissues (*p* < 0.0001). Notably, high C‐Myc expression was significantly correlated with both tumor grade (*p* = 0.003) and tumor stage (*p* = 0.005). ROC curve analysis demonstrated that the areas under the curve (AUCs) for C‐Myc, hsa‐miR‐1304, and hsa‐miR‐4652 were 0.99, 0.99, and 0.95, respectively (*p* < 0.0001), indicating strong diagnostic potential.

**Conclusion:**

These findings suggest that the upregulation of miR‐1304 and miR‐4652 could be used as biomarkers in OSCC. However, more studies with large samples are necessary.

**Abbreviations**
ANOVA: Analysis of variance
AUC: Area under the curve
cDNA: Complementary DNA
CI: Confidence interval
DAVID: Database for Annotation, Visualization, and Integrated Discovery
DFS: Disease‐free survival
GO: Gene Ontology
KEGG: Kyoto Encyclopedia of Genes and Genomes
lncRNA: Long noncoding RNA
miRNAs: microRNAs
ncRNA: noncoding RNA
NPCs: Neural progenitor cells
NSCLC: Non‐small cell lung cancer
OSCC: Oral squamous cell carcinoma
qRT–PCR: quantitative reverse transcription–polymerase chain reaction
ROC: Receiver operating characteristic
SNP: Single‐nucleotide polymorphism

## 1 Introduction

Tumors in the head and neck region represent approximately 5% of all tumors, with nearly half originating specifically in the oral cavity [1]. Among these, oral squamous cell carcinoma (OSCC) accounts for nearly 90% of cases and remains the most common malignancy of the oral cavity, with over 500 000 new cases diagnosed worldwide each year [2, 3]. Several symptoms may indicate OSCC, most commonly a non‐healing ulcer on the tongue, floor of the mouth, or inner cheek. These lesions may cause discomfort but can also remain asymptomatic in some cases [4].

Although surgical techniques and adjuvant treatments have improved over time, the clinical outcome of patients with OSCC is still largely determined by the underlying molecular and genetic alterations that drive tumor progression and recurrence [5]. Metastasis in advanced OSCC often occurs following a prolonged clinically undetectable phase of the disease and typically manifests after tumor recurrence. Therefore, a deeper understanding of the tumor microenvironment and clarification of its molecular profile are of great importance for early diagnosis and effective cancer therapy [6].

Therefore, in light of the high frequency of late‐stage diagnosis and the limitations of current clinical and histopathological approaches, there is an urgent need to identify reliable diagnostic molecular biomarkers, particularly those involved in gene regulatory networks, to facilitate early detection, improve risk stratification, and ultimately enhance clinical outcomes in oral squamous cell carcinoma [7].

MicroRNAs (miRNAs) are a class of small non‐coding RNA molecules that regulate gene expression by complementary base pairing with target mRNAs, leading to gene silencing through either mRNA degradation or translational repression [8]. As regulatory transcripts, microRNAs participate in the biogenesis of various neoplasms [9] with the potential to alter tumor phenotypes through their expression regulation [10]. Recent studies have highlighted that specific microRNAs in oral inflammatory conditions, playing critical roles in modulating immune responses and maintaining epithelial homeostasis in oral cavity [11]. Dysregulation of microRNAs (miRNAs) can disrupt the normal transcriptional landscape, leading to aberrant gene expression and contributing to malignant transformation in oral squamous cell carcinoma through modulation of critical oncogenic and tumor‐suppressive pathways [12]. Furthermore, these aberrantly expressed microRNAs have been associated with the initiation and progression of oral pathologies, suggesting their potential as early biomarkers for oral disease and targets for therapeutic intervention [13]. Certain microRNAs are closely correlated with the progression of OSCC [14]. miR‐31 identifide as an oncogenic factor in the tumorigenesis and progression of OSCC, whereas Li et al. demonstrated that the overexpression of miR‐10b led to enhanced OSCC invasion, migration, and activation of autophagic proteins [15]. The unique expression patterns of these molecules render them ideal candidates as diagnostic biomarkers and potential targets for pharmacological inhibitors [16]. mRNAs, microRNAs, long noncoding RNAs (lncRNAs), and circular RNAs influence the occurrence and development of cancer by regulating the C‐Myc gene [17] which has multifaceted regulatory roles in various aspects of transformation [18]. Accumulating evidence indicates that dysregulation of microRNAs (miRNAs) contributes to carcinogenesis by modulating oncogenes and tumor suppressor pathways. Aberrant expression of miR‐1304‐5p has been reported in several malignancies and is linked to tumor proliferation, migration, and metabolic reprogramming. Likewise, miR‐4652‐3p has been associated with epithelial–mesenchymal transition (EMT), apoptosis resistance, and cancer progression [19, 20, 21]. Additionally, the oncogenic transcription factor MYC is frequently overexpressed in various cancers and regulates genes involved in cell cycle progression, metabolism, and cellular transformation. It remains unclear whether C‐Myc overexpression initially drives metabolic changes, another hallmark of cancer, induced by transformation or if its common overexpression results from complex metabolic alterations that occur during malignant cell transformation [22].

Understanding the expression patterns of miR‐1304‐5p and miR‐4652‐3p, along with their relationship to C‐Myc, is crucial because dysregulated miRNAs can drive OSCC progression and may serve as early diagnostic molecular biomarkers or therapeutic targets. Clarifying these molecular interactions have the potential to enhance diagnostic accuracy and inform individualized therapeutic decisions in patients with OSCC. Additionally, precise knowledge of these associations may help identify patients at higher risk for aggressive disease and inform clinical decision‐making.

Although several microRNAs have been implicated in OSCC progression, the specific role of miR‐1304‐5p and miR‐4652‐3p in OSCC remains largely unexplored. Moreover, their potential interaction with key oncogenic regulators such as C‐Myc and their association with clinicopathological parameters have not been sufficiently clarified. This study aimed to identify the differential expression of miR‐1304‐5p and miR‐4652‐3p in OSCC patients. Moreover, we investigated whether the expression of miRNAs and the candidate miRNA target gene C‐Myc was associated with the clinicopathological characteristics and diagnostic potential of these molecular biomarkers in patients with OSCC.

## 2 Methods

### 2.1 Patient Tissue Samples

A total of 60 tissue samples (30 OSCC tumors and 30 matched adjacent non‐tumor oral epithelial tissues) were harvested from 30 patients diagnosed with oral squamous cell carcinoma from patients who underwent surgical treatment at Imam Khomaini Hospital, Iran, between October 2018 and December 2022. OSCC may present in various clinical forms, such as ulcerative, exophytic, or papillomatous lesions. In this study, only patients presenting with the ulcerative clinical form of OSCC were included. Informed consent, as a basic safeguard, was obtained from all participants, and Informed consent in writing was obtained in compliance with the guidelines of the Imam Hospital Institutional Review Committee. Resected OSCC tumors along with nearby normal mucosal tissues were collected from surgically treated patients and immediately preserved in liquid nitrogen at −80°C until RNA extraction. Normal mucosal tissue samples were obtained from regions located more than 10 cm away from the cancerous tissue. The inclusion criteria comprised patients with histopathologically confirmed primary OSCC, including both male and female patients, no prior chemotherapy or radiotherapy before surgery, sufficient tumor tissue available for molecular analysis, and complete clinicopathological data. The exclusion criteria included patients with recurrent OSCC, a history of other malignancies, previous neoadjuvant therapy, metastatic disease at diagnosis, inadequate tissue samples, incomplete clinical records, or lack of patient consent. The study cohort included both male and female patients with a mean age of 55 years (range: 24–86 years). Patient demographic information, including age, sex, and cancer stage, was retrieved from the patient records (Table 1).

**TABLE 1:** Association of miR‐4652, miR‐1304, and *C‐Myc* target gene expression with clinicopathological characteristics in OSCC.

Clinical features	Case no. (%)	*C‐Myc*		miR‐1304		miR‐4652	
		Mean ± SD	*p* value	Mean ± SD	*p* value	Mean ± SD	*p* value
Gender							
Female	6	5.3 ± 0.2	0.488	0.00 ± 0.49	0.280	0.025 ± 0.490	0.893
Male	24						
Age (Years)							
≥ 40	26	10.31 ± 5.91	0.244	0.00 ± 0.29	0.870	0.039 ± 0.49	0.47
< 40	4					0.34 ± 0.55	
Size(cm)							
≥ 5	19	11.4 ± 1.25	0.515	0.024 ± 2.53	0.734	0.025 ± 2.53	0.749
< 5	11			0.20 ± 0.35			
Vascular invasion							
Present	6	4.39 ± 0.8	0.845	0.00 ± 0.456	0.782	0.102 ± 0.456	0.750
Absent	24						
Lymphatic invasion							
Present	5	4.102 ± 0.5	0.112	0.00 ± 1.24	0.273	0.10 ± 1.24	0.239
Absent	25						
Necrosis Presence							
Present	7	4.16 ± 0.74	0.649	0.001 ± 0.927	0.789	0.15 ± 0.22	0.437
Absent	23					0.22 ± 0.33	
Stage							
Low (I and II)	6	8.49 ± 1.09	**0.007**	0.00 ± 1.09	0.505	0.036 ± 1.09	0.505
High (III and IV)	24						
Grade							
I	16	8.954 ± 5.8	0.187	0.00 ± 1.04	0.580	0.036 ± 1.04	0.584
II	13						
Status unknown	1						

*Note:* Bold indicates statistically significant value.Abbreviation: SD: Standard deviation.

### 2.2 RNA Extraction and cDNA Synthesis

High‐quality RNA was obtained from tissues using TRIzol (Invitrogen, Carlsbad, CA, USA) following the manufacturer's directions [23]. The RNA was dissolved in 30 mL of DEPC water (nuclease‐free water) and stored at −80°C for later use. The purity and quantity of the extracted RNA were measured via 1.5% (w/v) agarose gel electrophoresis and a Nanodrop spectrophotometer (NanoDrop Technologies, Wilmington, DE). All extracted RNA was normalized to 2 μg and and subsequently reverse‐transcribed into complementary DNA (cDNA) using the RevertAid First Strand cDNA Synthesis Kit (Thermo, Lithuania). For microRNAs, first, Linear RNA species were removed from total RNA using RNase R digestion at 37°C for 15 min, and then, cDNA was synthesized with random hexamers via a Reverted First Strand cDNA Synthesis Kit (Thermo, Lithuania).

### 2.3 RT‐qPCR Analysis

Quantitative real‐time PCR following reverse transcription, also called qRT–PCR, was used to determine the expression levels of the miR‐1304‐5p, miR‐4652‐3p, and C‐Myc genes. The reverse transcription products were subjected to amplification via SYBR Green Master Mix (Roche Co. Ltd.) in Exicycler TM 96 (Bioneer, Daejeon, Korea). The cycling protocols started with initial 5‐min denaturation at 95°C, followed by 40 cycles with steps at 95°C (10 s), 60°C (30 s), and 72°C (20 s). The relative expression levels of C‐Myc and microRNAs were normalized to those of the beta‐actin (ACTB) and U6 housekeeping genes, respectively. The relative expression levels of C‐Myc and microRNAs were calculated via the ^2 − ΔΔCt^ method relative to those of the housekeeping genes. The proper primers used in the present search were designed via Primer3plus software and are listed in Table 2.

**TABLE 2:** List of primers used for RT‐qPCR.

Target transcript	Primer type	Sequence (5′ → 3′)
miR‐1304‐5p	Forward	UUUGAGGCUACAGUGAGAUGUG
	Reverse	UCACAACCUCCUAGAAAGAGUAGA
miR‐4652‐3p	Forward	GCGGTTCTGTTAACCCATCCCCTCA
	Reverse	AGTGCAGGGTCCGAGGTATTC
*C‐Myc*	Forward	ATGCCCCTCAACGTT
	Reverse	TACGGGGAGTTGCAA
ACTB	Forward	GATCAAGATCATTGCTCCTCCTG
	Reverse	CTAGAAGCATTTGCGGTGGACC
U6	Forward	GCTTCGGCAGCACATATACTAC
	Reverse	CGCTTCACGAATTTGCGTGTCAT

### 2.4 Bioinformatics Analysis

The potential roles of miR‐4652 and miR‐1304 in carcinogenesis were explored by identifying candidate targets via the TargetScan target prediction online tool (http://www.targetscan.org). The functional annotation of the identified target genes was conducted via the Database for Annotation, Visualization, and Integrated Discovery (DAVID, version 6.7; https://david.ncifcrf.gov), which offers a comprehensive array of biological annotations to elucidate the functional significance of gene lists. Functional enrichment and pathway mapping analyses were conducted using the Gene Ontology (GO) and the KEGG (KEGG) databases to explore the biological processes and signaling pathways potentially regulated by the predicted target genes of hsa‐miR‐1304 and hsa‐miR‐4652.

### 2.5 Statistical Analysis

Statistical analyses and graphical illustrations were performed via SPSS 16.0 (SPSS Inc., Chicago, IL) and GraphPad Prism 8 (GraphPad Software, California, USA). The distribution of gene expression values was evaluated for normality with Kolmogorov–Smirnov and Shapiro–Wilk tests. To compare gene expression levels between tumor tissues and their matched adjacent normal tissues, paired statistical tests were applied: the paired Student's *t*‐test was used for normally distributed data, whereas the Wilcoxon signed‐rank test was used for nonnormally distributed data. Associations between gene expression levels and clinicopathological features were evaluated via appropriate parametric or nonparametric statistical tests on the basis of data distribution. Additional analyses included univariate and multivariate tests, bivariate statistics such as nonparametric tests, correlation analysis, and analysis of variance (ANOVA), linear regression for numerical outcome prediction, and Receiver operating characteristic curves were used to assess the diagnostic accuracy of gene expression markers. Results with *p*‐values less than 0.05 were considered significant.

## 3 Results

### 3.1 miR‐4652 and miR‐1304 Expression Is Upregulated in OSCC

**FIGURE 1:**
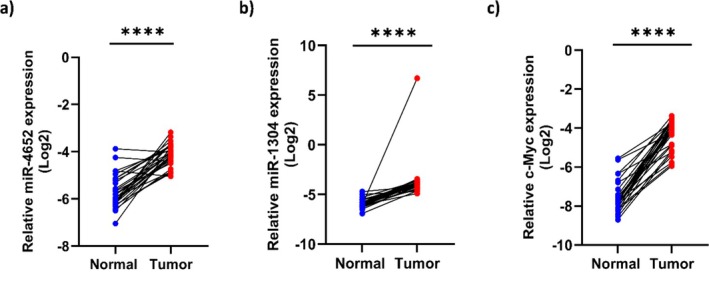
Quantitative RT–PCR analysis of (a) miR‐4652, (b) miR‐1304 and (c) *C‐Myc* levels in OSCC samples compared to neighboring normal tissues (*n* = 30). Expression data were normalized to U6 and ACTB expression. ****: *p* value < 0.0001.

The expression levels of miR‐4652 and miR‐1304 were detected in OSCC (*n* = 30) compared with their adjacent non‐tumoral tissues via RT–qPCR and normalized to U6 small nuclear RNA. The results revealed that the expression levels of miR‐1304 and miR‐4652 were significantly greater in cancer tissues than in normal tissues (*p* < 0.0001; Figure 1a,b). Furthermore, the expression of *C‐Myc* was significantly greater in OSCC tissues than in normal oral tissues (*p* < 0.0001; Figure 1c).

### 3.2 Associations Between miR‐4652, miR‐1304 and *C‐Myc* Expression Levels and Clinicopathological Features in OSCC

In this study, we analyzed the associations between the expression of miR‐4652, miR‐1304, and the *C‐Myc* target gene and the clinicopathological features of OSCC patients (Table 1).

Our findings indicate that there was no significant association between the levels of miR‐4652 or miR‐1304 and clinicopathological features, including stage, age, tumor size, sex and tumor necrosis (*p* > 0.05). A significant correlation was identified between the level of *C‐Myc* upregulation and tumor grade (*p* = 0.007). To further assess the diagnostic value of the *C‐Myc* gene in early‐stage oral cancer, we evaluated the relative expression of *C‐Myc* in 30 patients with early‐stage oral cancer (TNM stage I‐II) and 30 healthy controls. Our results revealed a significant association between increased C‐Myc expression in oral cancer and tumor stage (*p* = 0.005).

### 3.3 Potential Diagnostic Value of miR‐4652, miR‐1304 and C‐Myc in OSCC

**FIGURE 2:**
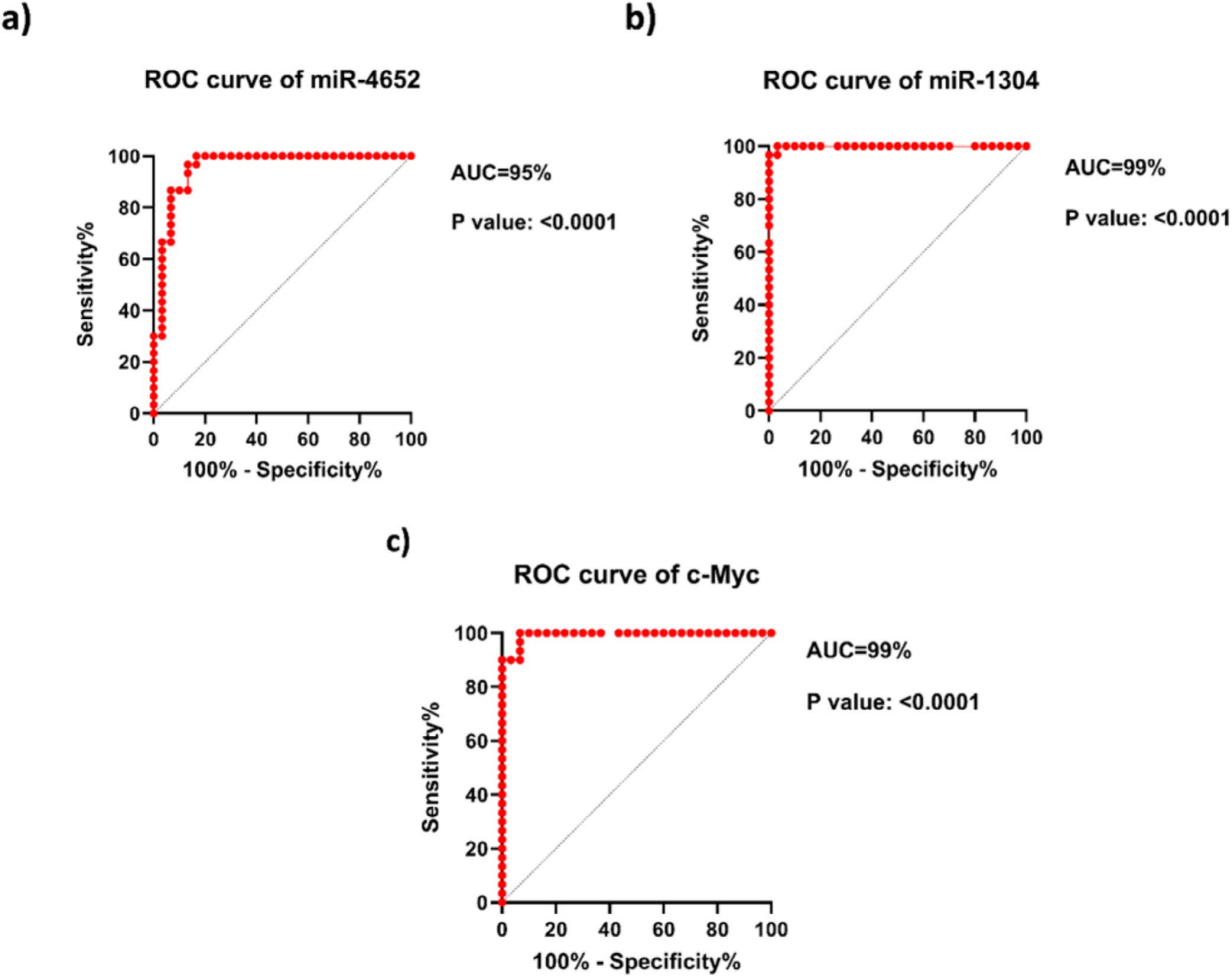
ROC curve analyses related to the expression of (a) miR‐4652, (b) miR‐1304, and (c) *C‐Myc* to discriminate OSCC tissues from adjacent normal tissues.

For the assessment of the diagnostic potential of miR‐4652, miR‐1304, and c‐Myc expression levels in OSCC samples contrasted with corresponding normal tissues, ROC curve analysis was performed (Figure 2a–c). The best threshold points were identified through the Youden index, and the area under the ROC curve (AUC), sensitivity, and specificity were subsequently calculated.

ROC analysis revealed AUC values of 95% for miR‐4652, 99% for miR‐1304, and 99% for c‐Myc (*p* < 0.0001), indicating their excellent diagnostic performance in distinguishing tumor tissues from adjacent normal tissues.

The optimal cutoff values, sensitivity, specificity, AUC, and the associated 95% CIs for each biomarker are displayed in Table 3.

**TABLE 3:** ROC curve characteristics of miR‐4652, miR‐1304, and c‐Myc.

Gene	Optimal cutoff (∆CT)	Sensitivity (%)	Specificity (%)	AUC (%)	CI95%	*p*
miR‐4652	< 4.930	96.67	86.67	95	0.9025 to 1.000	< 0.0001
miR‐1304	< 4.695	96.67	100	99	0.9952 to 1.000	< 0.0001
c‐Myc	< 6.133	100.0	93.33	99	0.9809 to 1.000	< 0.0001

Abbreviations: AUC: area under the curve, CI: confidence interval.

## 4 Discussion

Approximately 90% of people with an average risk of oral cancer are diagnosed with squamous cell carcinoma, which originates from the epithelial lining of the oral cavity [24]. Recent studies and compelling evidence have revealed that the aberrant expression of miRNAs in human cancer depends on genetic and chromosomal abnormalities, epigenetic alterations, and single‐nucleotide polymorphisms [25]. Emerging evidence has suggested that miRNAs are abnormally expressed in tumor tissues and are highly associated with the the initiation and development of different disorders and cancers, including oral [26]. miR‐4652 and miR‐1304 are among the numerous miRNAs studied in cancers and play widely important roles in many different cellular mechanisms of cancer formation and progression [27, 28].

In the present study, we demonstrated significant upregulation of miR‐1304‐5p, miR‐4652‐3p, and C‐Myc in OSCC tissues compared with adjacent normal tissues. In a specific study, miR‐1304 was reported to be upregulated in breast cancer, and patients with high miR‐1304 expression exhibited shorter disease‐free survival (DFS) [19]. Cheng‐Gang Li et al. reported that miR‐1304 functions as a tumor suppressor in NSCLC, as ectopic overexpression markedly suppressed cellular proliferation and clonogenic capacity, and concurrently induced apoptosis and cell‐cycle arrest [29]. In contrast, miR‐4652 is downregulated in NSCLC, indicating its potential role in tumor progression [27].

However, in NSCLC tissues miR‐1304 expression was found to be downregulated, and experimental suppression of miR‐1304 in NSCLC cell lines enhanced cell growth and viability, indicating its tumor suppressor role [30]. Lu and colleagues reported that elevated miR‐1304 expression correlates with larger tumor size, poorer differentiation, and more advanced stages of esophageal cancer, indicating its potential as a biomarker for diagnosis and prognosis [28]. In nasopharyngeal cancer, increased expression of miR 4652 3p promotes cell invasion and metastasis, and this effect is exerted by targeting the HIPK2 gene, a potential tumor suppressor. SPEN (or SHARP) protein, as an RNA‐binding transcriptional regulator, increases miR 4652 3p expression by activating the PI3K/AKT/cJUN pathway. The increase in this microRNA inhibits HIPK2, thereby stimulating the processes of EMT, migration, invasion, and metastasis. Therefore, SPEN plays a key role in the progression of nasopharyngeal cancer as an upstream regulator through the induction of miR‐4652 3p and the suppression of HIPK2 [21, 31]. Additionally, bioinformatic analyses revealed that miR‐4652‐3p and miR‐1304 are upregulated in head and neck squamous cell carcinoma (HNSCC) and may serve as potential prognostic markers across different HNSCC subtypes [32, 33]. These findings are consistent with previous reports, as we observed significant upregulation of miR‐1304‐5p and miR‐4652‐3p in OSCC tissues, supporting their potential roles in tumor progression and the pathogenesis of oral squamous cell carcinoma. Our results align with prior studies showing that dysregulated miRNA expression contributes to OSCC development, highlighting the possible oncogenic involvement of these two miRNAs. Several other miRNAs have also been reported to be aberrantly expressed in OSCC and are implicated in tumor progression and malignant behavior [34]. These findings are consistent with previous reports, as our study demonstrated a significant upregulation of miR‐1304‐5p and miR‐4652‐3p in OSCC tissues, supporting their potential roles in the progression and pathogenesis of oral squamous cell carcinoma. Despite the marked increase in the expression levels of miR‐1304‐5p and miR‐4652‐3p, no statistically significant association was observed between their expression and clinicopathological parameters.

A growing amount of evidence indicates that C‐Myc serves as a target gene for miR‐1304 and miR‐4652, which inhibit C‐Myc expression, influencing cell proliferation, migration, and invasion in various human cancers [27, 35]. C‐Myc, L‐myc, and N‐myc belong to the MYC proto‐oncogene family, encodes nuclear phosphoproteins critical for cell proliferation and transformation [36]. In oral cancers, elevated C‐Myc expression has been consistently reported [37], with gene expression analyses demonstrating its involvement in most human cancers, and Marconi et al. further confirming its significant overexpression in an OSCC cell model, highlighting its central role in tumor development and progression [38]. C‐Myc, a key gene in oral cancer, is regulated by multiple microRNAs (miR‐145, miR‐1294, miR‐let‐7a, and miR1254), which play a role in tumor growth and metastasis [39, 40, 41]. It is important to emphasize that C‐Myc functions as a master transcriptional regulator that coordinates proliferation, metabolism, and multiple hallmarks of malignancy; its activation can therefore drive aggressive tumor behavior and modulate the tumour microenvironment [42, 43]. Importantly, C‐Myc both influences and is influenced by non‐coding RNAs: for example, miR‐1304 has been linked to MYC regulation in cancer contexts (circ‐PRMT5/miR‐1304/MYC axis), and miR‐4652‐3p has been reported as an SPEN‐induced oncogenic miRNA with downstream effects on pathways that intersect MYC signalling and metabolism [21, 44]. This evidence highlights the relevance of C‐Myc and that microRNAs have the potential to be used as biomarkers for oral cancer diagnosis and therapy [38, 45]. Additionally, accumulating evidence suggests that oxidative stress, often triggered by chronic inflammation, has a central role in OSCC development and progression [46, 47]. Reactive oxygen species can induce DNA damage, promote mutagenesis, and alter the expression of miRNAs and oncogenes such as C‐Myc. Considering the interplay between oxidative stress and inflammatory pathways could further clarify the molecular mechanisms underlying OSCC and may provide additional targets for therapy or early diagnosis [48, 49].

The present study demonstrated that miR‐1304‐5p and miR‐4652‐3p are significantly upregulated in OSCC tissues, coinciding with C‐Myc overexpression. While some reports in other cancer types suggest that these miRNAs can target and inhibit C‐Myc, their simultaneous upregulation in OSCC implies that a direct inhibitory effect is unlikely or not dominant in this tissue context. Given the context‐dependent nature of miRNA function and their ability to modulate both oncogenes and tumor suppressors, it appears that miR‐1304‐5p and miR‐4652‐3p operate within a complex regulatory network in OSCC. These miRNAs may be influenced by activated oncogenic pathways and concurrently contribute to the tumor‐promoting molecular program.

Understanding the underlying mechanisms provides deeper insights into OSCC progression. C‐Myc, as a key regulator of cell proliferation, metabolism, and malignant behavior, plays a central role in tumor development. The aberrantly regulated miR‐1304‐5p and miR‐4652‐3p may modulate these pathways, thereby promoting tumor growth and invasion.

We statistically analyzed the associations of C‐Myc expression with clinicopathological factors and revealed positive correlations with tumor size, lymph node metastasis, histology, age, perineural invasion, lymphovascular invasion, and invasion depth. Similarly, previous studies have reported increased C‐Myc expression in advanced tumors and its correlation with tumor stage and grade [37, 50]. Univariate analysis further examined the combined effect of clinicopathological factors and myc expression on mortality risk, revealing a positive correlation between C‐Myc expression, stage, and grade [51].

ROC curve analysis demonstrated that all three evaluated biomarkers, including miR‐4652, miR‐1304, and c‐Myc, showed excellent diagnostic performance in distinguishing OSCC tissues from adjacent normal tissues. The AUC for miR‐4652 was 0.95, with a sensitivity of 96.67% and a specificity of 86.67%. Both miR‐1304 and c‐Myc exhibited superior diagnostic accuracy, with an AUC of 0.99. Specifically, miR‐1304 showed 96.67% sensitivity and 100% specificity, whereas c‐Myc demonstrated 100% sensitivity and 93.33% specificity, indicating a high discriminatory capacity between tumor and normal samples.

Such a level of diagnostic accuracy is rarely achieved by individual molecular biomarkers and highlights the considerable potential of these miRNAs and c‐Myc as reliable candidates for early detection and risk stratification of oral squamous cell carcinoma. However, despite these promising findings, the results should be interpreted with caution and further validated in larger independent cohorts prior to clinical application.

## 5 Conclusion

In conclusion, our findings indicate that the upregulated expression of hsa‐miR‐1304 and the novel hsa‐miR‐4652, along with elevated C‐Myc levels in OSCC patients, may serve as promising molecular biomarkers for the early detection and monitoring of oral squamous cell carcinoma. The significant association between C‐Myc expression and clinicopathological features such as tumor grade and stage further highlights its potential role in disease progression. Moreover, the high diagnostic accuracy demonstrated by ROC curve analysis underscores the potential clinical utility of these biomarkers in improving OSCC screening strategies. Nevertheless, further large‐scale, population‐based studies are required to validate these findings and to elucidate the underlying molecular mechanisms involved in OSCC pathogenesis before their implementation in routine clinical practice.

## Author Contributions

**Sara Bagheri Farahani:** investigation, formal analysis, writing – review and editing, writing – original draft. **Ehsan Keramati:** investigation, writing – original draft, writing – review and editing. **Marziyeh Etesami:** formal analysis, writing – review and editing, writing – original draft. **Faranak Jamshidian:** conceptualization, data curation, formal analysis, writing – original draft preparation, and writing – review and editing.

## Funding

The authors have nothing to report.

## Ethics Statement

All procedures in this study adhered to the principles of the Declaration of Helsinki and were approved by the Ethics Committee of the Islamic Azad University, Science and Research Branch (Ethical Code: IR.IAU.SRB.REC.1399.094).

## Consent

Each participant voluntarily provided informed consent prior to inclusion in the study.

## Conflicts of Interest

The authors declare no conflicts of interest.

## Data Availability

The data that support the findings of this study are available from the corresponding author upon reasonable request.
